# AI-assisted phenotyping in a zebrafish hypophosphatasia model enables early and precise detection of skeletal alterations

**DOI:** 10.1038/s41598-025-19199-w

**Published:** 2025-09-17

**Authors:** Regina Hark, Simon Zürlein, Viet T. Nguyen, Gunther Gust, Lukas Hekel, Daniel Liedtke

**Affiliations:** 1 Institute of Human Genetics, Am Hubland, Biocenter, Julius-Maximilians-University Würzburg, 97074 Würzburg, Germany; 2 Chair for Enterprise Artificial Intelligence, Center for Artificial Intelligence and Data Science (CAIDAS), Sanderring 2, Würzburg, Germany

**Keywords:** Hypophosphatasia, Zebrafish, *ALPL*, Deep learning, Vision Transformers, Phenotype classification, Explainable AI, Disease model, Data processing, Metabolic bone disease

## Abstract

Hypophosphatasia (HPP) is a rare genetic disorder mainly affecting bone and tooth mineralization in patients due to *ALPL* gene mutations. Understanding genotype-phenotype correlations in HPP remains challenging due to different severities and the disease’s heterogeneity. To address this, we established a novel zebrafish animal model (*alpl*^wue7^), which mimics severe HPP disease forms. To bypass limitations in human-based phenotypic classification of skeletal alterations in this transgenic line, we developed and trained an artificial intelligence (AI) model capable of image-based classification with 68% accuracy—an improvement of 79% over manual classification. Our AI model could successfully identify early developmental alterations independent of altered image magnification, coloration quality and executing scientists. Using attention rollout, we further visualized AI decision-making, revealing not only expected focus on early bone structures but also unexpected emphasis on the otoliths—parts of the zebrafish’s hearing and balancing organ. We see applications of our AI system in analyzing other skeletal disorder models as well as in providing an unbiased, high-throughput phenotypic rescue quantification assay for potential drug screening applications in zebrafish larvae. Overall, our findings establish an integrated platform for studying HPP and open new avenues for AI-assisted phenotyping and therapeutic discovery.

## Introduction

Hypophosphatasia (HPP) is a rare, genetically inherited metabolic disorder that manifests in different clinical forms and severities^1,2^. It is classified into maximum six clinical subtypes, which exhibit highly variable symptoms. These range from the perinatal lethal form as the most severe, to the adult form, which represents the mildest manifestation (adult: OMIM # 146300, infantile: OMIM # 241500, childhood: OMIM # 241510, odontoHPP: OMIM # 146300)^3^. The most prominent symptoms of all HPP patients are caused by impaired bone and tooth mineralization^4^, which subsequently results in increased occurrence of fractures, ricket-like deformities, osteomalacia, short stature, and premature tooth loss^2^. The estimated incidence of severe HPP is approximately 1 in 300,000 births in Europe^5^ and 1 in 100,000 in North America^6^. In addition to skeletal symptoms, HPP can also present with milder manifestations in other organs, including the liver, kidneys, lungs, and central nervous system^4,7^.

The genetic cause of hypophosphatasia (HPP) has been causatively linked to loss-of-function mutations in the *ALPL* gene (HGNC ID:438)^8–10^, which encodes the ectoenzyme ’Tissue Nonspecific Alkaline Phosphatase’ (TNAP; UniProt ID: P05186). Disease-causing variants in *ALPL* can be inherited in either an autosomal recessive or autosomal dominant manner^11^. The most severe forms of HPP are typically observed in individuals who are compound heterozygous or homozygous variant carriers, resulting in a complete loss of TNAP function^12^. TNAP plays a central biochemical role in bone mineralization by regulating the formation of hydroxyapatite^13^. It does so through the dephosphorylation of mineralization inhibitors such as inorganic pyrophosphate (PPi) and phosphorylated osteopontin (pOPN), thereby facilitating the deposition of hydroxyapatite crystals in the extracellular matrix (ECM)^14,15^. During skeletal development, both the concentration and enzymatic activity of TNAP in the ECM are critical, as they precisely control the local balance of substrates and products necessary for proper mineralization of skeletal tissues^2,13^.

Several studies have attempted to establish genotype–phenotype correlations in HPP; however, the exceptional heterogeneity of the disease has made this challenging. As a result, many *ALPL* genetic variants still cannot be reliably associated with a specific phenotype or clinical outcome^12,16,17^. Furthermore, identical *ALPL* variants can lead to various HPP phenotypes in intra-familiar patients. So far, this observation cannot be satisfactorily explained and hints to additional confounding factors during disease progression^17,18^.

To identify such potential factors and investigate the molecular mechanisms underlying HPP, several mouse models have been developed^19^. However, similar to the Heterogeneity observed in patients, mouse models mimicking the infantile HPP subtype exhibit inconsistent phenotypes. For example, only approximately 50% of homozygous mice display impaired bone mineralization eight days after birth^20^. As an auxiliary vertebrate model for studying bone development, the zebrafish (*Danio rerio*) has been widely and successfully used to model various human diseases^21^. Initial studies from our lab using *alpl* morpholino knockdown and Tnap chemical inhibition in zebrafish indicated prominent effects on earliest zebrafish bone development^22^. However, morpholino-based knockdown can lead to phenotypes that differ from those caused by genetic ablation due to genetic compensation mechanisms^23,24^, while chemical inhibition can result in pharmacological off-target effects^25^.

To eliminate potential misinterpretations caused by genetic compensation or off-target effects, we generated a stable transgenic *alpl* knockout zebrafish line lacking both the *alpl* promoter and exon 1 (*alpl*^wue7^). This model was histologically investigated for common HPP-related phenotypes and exhibited mineralization defects across multiple skeletal structures, consistent with findings from previous knockdown studies^22^.

Given the subtle and heterogeneous disease presentations observed in our zebrafish HPP model, manual evaluation of skeletal structures—despite high-resolution imaging—proved to be both subjective and error-prone. These limitations underscored the need for a more robust, scalable, and unbiased approach to phenotype classification. To overcome these challenges, we turned to automated image analysis using deep learning, a methodology that has rapidly gained prominence in biological and medical research. Convolutional neural networks (CNNs) have long been the dominant architecture in image-based classification tasks within these fields^26–31^. In this study, we employed Vision Transformers (ViTs)^32^, a state-of-the-art model architecture for image classification. ViTs offer advantages over CNNs in capturing global image features and have demonstrated strong performance in tasks involving limited data and subtle visual cues—such as biological phenotypes. We specifically used the BEiT (Bidirectional Encoder representation from Image Transformers) framework^33^, which builds upon the ViT architecture. To adapt the model to our biological image data, we applied a transfer learning (see subsection 4.3) approach, initializing with a BEiT model pre-trained on the ImageNet-1K dataset—a large-scale image collection containing approximately one million natural images^34^.

To address the unresolved genotype–phenotype correlations in HPP, we apply state-of-the-art deep learning techniques to our newly developed *alpl*^wue7^ knockout zebrafish model. This approach enables unbiased classification of heterogeneous skeletal phenotypes and revealed subtle, previously unrecognized morphological alterations. By combining the AI model with a curated dataset of annotated images, we provide novel insights into the complex phenotypic manifestations of HPP and establish an explainable, scalable framework for early phenotypic analysis. These contributions advance the fields of HPP research, biomedical image analysis, and offer further applicability for drug screening and skeletal disease modeling across species.

## Methods

This section describes the acquisition and preprocessing of microscopy images (Sections 2.1 to 2.3), and the development and evaluation of the AI model (Sections 2.4 to 2.7). Detailed methods for the generation and characterization of the transgenic zebrafish *alpl*^wue7^ line are stated in supplementary sections 4.2.1–4.2.6. All procedures involving experimental animals were performed in compliance with local animal welfare laws (Tierschutzgesetz §11, Abs. 1, Nr. 1 and corresponding TierSchVersV, husbandry permit number 568/300-1870/13), European Union animal welfare guidelines (EU directive 2010/63/EU), and ARRIVE guidelines. Establishment and propagation of *alpl* transgenic lines has been approved by the Regierung von Unterfranken (permit number 55.2.2-2532-2-1472). All experimental zebrafish used in this study were euthanized until 120 hours post fertilization (hpf) by 10 min incubation in 0.3 mg/ml MS-222 on ice.

### Bone and cartilage double staining

For simultaneous visualization of cartilage and mineralized tissue, acid-free alizarin red and alcian blue staining was performed in zebrafish larvae at 120 hpf according to standard protocols^35^. PTU treated larvae were killed and fixed in 4 % paraformaldehyde/phosphate buffered saline (PBS) for 2 h at room temperature. A double staining solution consisting of 0.001 % Alizarin red S (m/v, C.I. 58005, Carl Roth, Germany) and 0.4 v/v % Alcian blue 8 GS (m/v, C.I. 74240, Carl Roth GmbH, Germany), 150 mM MgCl_2_ diluted in 70 % ethanol was used subsequently. After staining for two days, larvae were destained in 20 % glycerol/1 % KOH/water for 2 h, and 50 % glycerol/1 % KOH/water overnight. Larvae were transferred in a rising dilution series of 50 %, 75 % glycerol/water (each step 5 min, RT) and were finally dissected in 100 % glycerol. The trunk was used for genotyping (see supplement method 4.2.5), while heads were manually dissected into viscerocranium (“DOWN”) and neurocranium (“UP”).

### Imaging

Microscopy images were acquired either with a Keyence BZ-X810 fluorescence microscope (used filter set: Texas Red, bright field) or a ZEISS Axio.Imager A1 microscope (camera: AxioCAM “MRc”, used filter set: Texas Red and bright field, fluorescent lamp: ZEISS HXP 120). Brightfield images were taken for assesment of general morphology, fluorescence imaging was only used to clarify red staining. Images were aquired correspondingly by Axiovision software (ZEISS, Germany), or BZ-X800 Analyzer (Keyence Corporation, Japan). Images were subsequently processed as .tif or .zvi formats by using ImageJ Fiji (https://fiji.sc/) and CorelDraw Graphics Suite 2023 software (Corel Corporation).

### Dataset

The dataset used in this study consists exclusively of brightfield microscopy images of bone cartilage staining, which were obtained in the experiments described in subsection 2.1). Each image was assigned an unique ID during preprocessing, encoding metadata related to the experiment and the individual specimen. No images were excluded due to irregularities. The ground truth labels were assigned based on genetic genotyping.

A total of 97 zebrafish larvae were examined, 36 of which were wildtype (*alpl*^+/+^) genotypes, 38 heterozygous (*alpl*^wue7/+^), and 24 homozygous (*alpl*^wue7/wue7^). The dataset includes neurocranium (ventral) and viscerocranium (dorsal) microscopy images captured at varying magnification levels (zoom) levels (see Table 1).

**Table 1:** Number of images per class for each craniofacial structure (viscerocranium (visc) and neurocranium (neuro)) and zoom level.

Structure	Zoom	Wildtype	Heterozygous	Homozygous
Visc	20$\times$	52	58	46
	10$\times$	69	73	69
	4$\times$	20	38	27
Neuro	20$\times$	48	43	52
	10$\times$	77	77	65
	4$\times$	22	42	23
Total		288	331	282

The raw images were initially acquired in .tif or .zvi format at a resolution of 1920$\times$1440 pixels. To ensure a uniform format for processing, all images were converted to .png using the ImageJ python library^36^ and subsequently compiled into the final dataset in .pkl format. Color information was fully preserved throughout these pre-processing steps.

The complete dataset supporting this paper has been made publicly available on Zenodo and can be accessed at https://zenodo.org/records/15269595.

### Foundational model architecture: BEiT

To classify the microscopy images, a Transformer-based model was used as the basis. The transformer architecture, introduced in “Attention Is All You Need”^37^, first revolutionized natural language processing (NLP) through the self-attention mechanism. Transformers are the foundation of popular large language models such as ChatGPT (Generative Pre-trained Transformer)^38^ or DeepSeek^39^. Their application to computer vision was later pioneered with the Vision Transformer (ViT)^32^, which divides images into smaller, grid-like regions (patches) and analyzes their interrelations - analogous to how language models capture dependencies between words.

BEiT extends the ViT architecture by introducing a self-supervised pretraining strategy. Self-supervised learning leverages the intrinsic structure of data to generate learning signals, eliminating the need for labeled examples. As part of this strategy, BEiT incorporates masked image modeling (MIM). The objective of MIM is to enable the model to infer missing visual information based on the surrounding context. During pretraining, portions of the input image are randomly masked, and the model is trained to reconstruct these masked regions. This encourages the learning of meaningful and robust image representations^33^. For this pretraining, the ImageNet-1K^34^ dataset, containing approximately 1.2 million images at a resolution of $224 \times 224$^33^, is used.

Following the self-supervised pretraining, BEiT undergoes an additional refinement step at different resolutions on an auxiliary dataset. The model used in this work was subsequently retrained on the ImageNet-1K dataset, this time using the images ground truth classes, at a higher resolution of $512 \times 512$ pixels. Only after this intermediate pretraining step is a task-specific classification layer added, enabling the model to make predictions for the downstream task–in this case, identifying skeletal features in histological images of zebrafish larvae. The BEiT-Large-Patch16-512 model, a specific variant of BEiT, represents the final architecture applied in this study. It processes images by first dividing them into fixed-size patches, then passing these through 24 transformer layers, and finally aggregating the learned representations in a classification head to produce the final prediction^33^.

### AI experimental setup

The experimental setup was designed to systematically train and evaluate AI classification models on microscopy images of zebrafish skeletal structures. The primary objective was to determine whether deep learning models can effectively distinguish between wildtype, heterozygous, and homozygous genotypes based on visual features, identifying minor changes at early developmental time points. The code used in the creation of this paper is available on GitHub (https://github.com/simonzrln/zebrafish_paper).

**Fig. 1:**
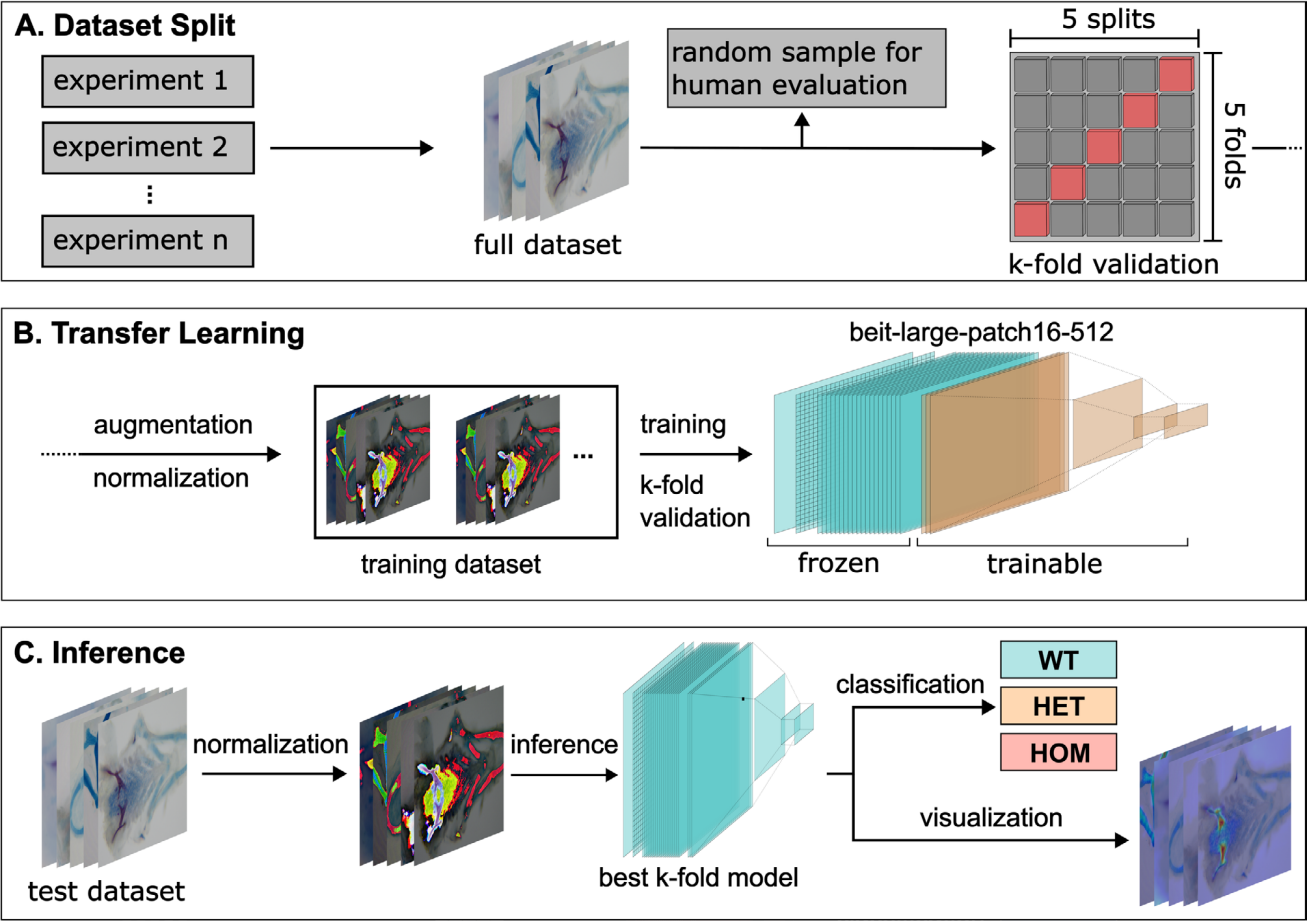
Overview of the AI-based classification pipeline. (**A**) Images from multiple experiments are collected. A subset of 100 images was selected for manual human classification. k-fold cross-validation is performed. (**B**) The dataset is augmented and normalized. The BEiT model is trained using k-fold cross-validation (**C**) Inference is performed on the corresponding test dataset. Images were classified into three genotype classes: wildtype (WT), heterozygous (HET) and homozygous (HOM). Attention Rollout was applied to visualize the models decision making.

To ensure an unbiased evaluation, the available images were first divided into two parts: a training set and a test set for assessing final performance. A stratified five-fold cross-validation approach was applied when splitting the data to ensure that each of the five subsets contained a balanced distribution of genotypes. To avoid overlap between training and validation sets, images from the same fish were assigned to a single split. The dataset was shuffled five times to ensure that each subset served as the test dataset once, resulting in five distinct training and test sets, as illustrated in Fig. 1A. To minimize variance resulting from unfavorable data partitioning, an additional stratified five-fold split was applied within the training set to create validation sets for tuning model parameters during training. After training, the model resulting from the best-performing fold, as determined by accuracy on the validation set, was used to classify the images in the test set.

As shown in Fig. 1B, the BEiT-large-patch16-512 model was adapted to the zebrafish skeleton images using transfer learning. The adaptation strategy is described in the Appendix (see subsection 4.4).

Furthermore Attention Rollout was used to improve the interpretability of the model’s decisions, highlighting image regions that most strongly influenced the model’s predictions^40,41^. Attention Rollout works by tracing how information flows through the layers of the transformer and combining the model’s internal focus on different image regions across all of its layers. In doing so, it estimates how much each image patch contributes to the final prediction. Regions that are considered important by the model are highlighted more strongly, while less relevant areas receive little or no emphasis. The resulting map reflects the importance of each image patch within the $16 \times 16$ grid structure of the Vision Transformer and was subsequently smoothed to create continuous heatmaps. These heatmaps were then overlaid onto the original images by combining of the original image with the heatmap (see Fig. 1C).

### Benchmarks

To evaluate the performance of the BEiT-based model, two key baselines were selected for comparison: (1) human classification performance and (2) alternative deep learning architectures commonly used in medical image analysis. These benchmarks provide a meaningful reference to assess the potential advantages and limitations of the BEiT model for the given classification task.

Human classification performance serves as an empirical baseline to compare AI-based predictions against assessments by four individuals. To evaluate human accuracy, a random sample of 100 images was drawn from the dataset. Each participant was assigned an individual sample of pictures. Participants classified the images using a graphical user interface. The interface allowed users to select among the three predefined genotypic classes.

In addition to the human benchmark, two deep learning architectures were selected for comparison. ResNet-101^42^ represents a well-established convolutional neural network (CNN) baseline, widely used in medical imaging due to its stability in training and strong performance across various classification tasks. It was chosen over simpler (e.g., ResNet-50) and more complex variants (e.g., ResNet-152), based on superior performance observed during preliminary testing. ViT-base-patch16-224-in21k^32^ was included as a Transformer-based benchmark to evaluate whether the masked image modeling pretraining of BEiT provides a significant performance advantage over standard Vision Transformers. As the underlying architecture of BEiT is based on ViT, this comparison isolates the effect of the pretraining strategy.

### Evaluation metrics

To assess the performance of the AI models, we later report several evaluation metrics including accuracy, precision, recall, sensitivity, F1-score, and the Area Under the Curve (AUC). These metrics provide a comprehensive analysis of classification performance across different aspects.

A prediction is considered correct if the class with the highest assigned probability matches the ground truth label. All metrics are computed as the average over all five test datasets, with the standard deviation reported to quantify variability in model performance. Additionally, statistical significance in performance differences between the AI model and human classification was assessed using a Student’s t-test.

**Accuracy** measures the proportion of correctly classified instances among all instances,

1 $$\begin{aligned} \text {Accuracy} = \frac{TP + TN}{TP + TN + FP + FN}, \end{aligned}$$

where *TP* (True Positives) and *TN* (True Negatives) represent correctly classified positive and negative samples, respectively, while *FP* (False Positives) and *FN* (False Negatives) denote misclassified samples.

**Precision** quantifies how many of the predicted positive instances are actually positive,

2 $$\begin{aligned} \text {Precision} = \frac{TP}{TP + FP}. \end{aligned}$$

**Recall**, also referred to as sensitivity, measures the proportion of actual positives that were correctly identified,

3 $$\begin{aligned} \text {Recall} = \frac{TP}{TP + FN}. \end{aligned}$$

**F1-Score** is the harmonic mean of precision and recall, providing a balanced measure between the two,

4 $$\begin{aligned} \text {F1-Score} = 2 \times \frac{\text {Precision} \times \text {Recall}}{\text {Precision} + \text {Recall}}. \end{aligned}$$

**Area Under the Curve (AUC)** represents the area under the Receiver Operating Characteristic (ROC) curve and evaluates the ability of the model to distinguish between classes. In multi-class classification, AUC is computed using a one-vs-all (OvA) approach, where each class is treated as the positive class while the remaining classes are considered negative^43^. This allows for separate evaluation of the model’s ability to differentiate each genotype.

AUC is formally defined as the integral of the True Positive Rate (TPR) against the False Positive Rate (FPR),

5 $$\begin{aligned} \text {AUC} = \int _{0}^{1} TPR(FPR) dFPR, \end{aligned}$$

where

6 $$\begin{aligned} \text {TPR} = \frac{TP}{TP + FN}, \quad \text {FPR} = \frac{FP}{FP + TN}. \end{aligned}$$

To assess whether the performance difference between AI models and human classification is statistically significant, a **Student’s t-test** is conducted. Given two sets of performance scores, the t-statistic is computed as

7 $$\begin{aligned} t = \frac{\bar{X}_1 - \bar{X}_2}{\sqrt{\frac{s_1^2}{n_1} + \frac{s_2^2}{n_2}}}. \end{aligned}$$

where $\bar{X}_1$ and $\bar{X}_2$ are the mean performance values of the AI model and human classification, respectively, $s_1^2$ and $s_2^2$ are the variances, and $n_1$ and $n_2$ are the number of observations in each group.

## Results

### Establishment and Investigation of *alpl* Knockout Zebrafish Line

**Fig. 2:**
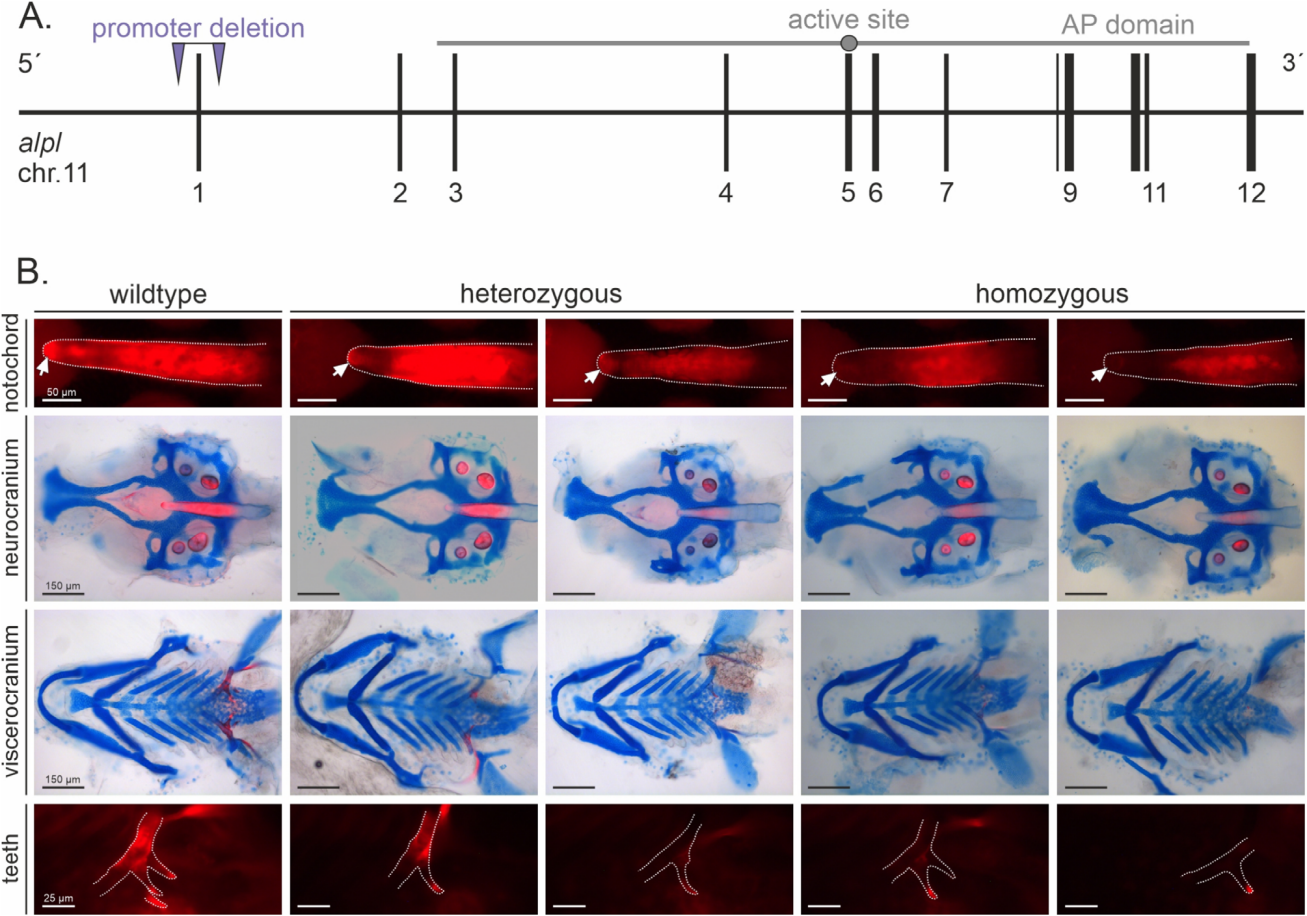
Generation and investigation of transgenic ***alpl*** knockout zebrafish line. (**A**) Schematic drawing of the genomic region on chromosome 11 deleted in *alpl*^wue7^ zebrafish larvae. The inserted *alpl* promoter deletion is including the *alpl* promoter region, together with exon 1 and parts of 5’UTR and intron 1. (**B**) Bone and cartilage double-stainings of 120 hpf transgenic zebrafish larvae display slightly changed craniofacial development in the neurocranium and viscerocranium. Bone (red) and cartilage (blue). Two individuals representing the same genotype, but different phenotype severities are shown for hetero- and homozygotes. wildtype = *alpl*^+/+^; heterozygous = *alpl*^wue7/+^; homozygous = *alpl*^wue7/wue7^.

For molecular investigation of HPP disease progression, we generated a new transgenic animal model in zebrafish. Initially, two sgRNAs targeting the *alpl* locus (ENSEMBL ID: ENSDARG00000015546) within the 5’UTR region (*alpl* c.-451_-431) and intron 1-2 (*alpl* c.37+494_37+514) were designed and generated by RNA *in vitro* transcription. Both sgRNAs were mixed in a 1:1 ratio, preassembled with Cas9 protein, and were injected into one cell-stage zebrafish eggs. CRISPR/Cas9 functionality was tested by gDNA sequencing of single, positively injected zebrafish embryos at 3 days post fertilization (dpf). After raising positively injected embryos to adulthood, individual crossing of F0 founder fish with *AB/AB* wildtype zebrafish were conducted. Identification of F1 embryos carrying a heterozygous deletion within the desired genomic region was done by gDNA-PCR amplification and gel electrophoresis (Figure S 1). Subsequent generation crossing of heterozygous *alpl*^wue7/+^ animals confirmed stable transgene propagation and appearance of homozygous *alpl*^wue7/wue7^ embryos. For the exclusion of possible CRISPR off target effects, we out-crossed our mutants to wildtype zebrafish to screen for phenotype continuity between generations. Mapping of the genomic position of the variant of *alpl*^wue7^ was confirmed by Sanger Sequencing and corresponds to *alpl* c.-506_37+78del in transcript ENSDART00000146461.3 (supplementary Figure S 1 B). The deleted region within the *alpl* gene locus is spanning 621 bp (GRCz11 genomic location: chr11:27968625-27969245), including 5’UTR sequences, the protein coding exon 1, as well as a small region in the of intron 1-2 (affected ENSEMBL Transcripts: alpl-202/ENSDART00000146461.3 (canonical reference) and alpl-203/ ENSDART00000147984.3 (predicted transcript); Fig. 2A. Since *alpl* exon 1 contains the transcriptional start site along with 37 bp of protein coding sequence and cis-elements of the promoter of the *alpl* mRNA transcript, this mutation is considered to result in no *alpl* mRNA expression and subsequently lack of Tnap protein. A statistically highly significant reduction of *alpl* mRNA expression in homozygous *alpl*^wue7/wue7^ larvae was confirmed via qPCR analysis (see supplement Figure S 3; pre-genotyped, pooled 120 hpf whole embryo cDNAs). Moreover, qPCR analyses did not show statistical significant changes in *alpl* expression in heterozygous samples. Thus, we concluded the successful generation of a transgenic zebrafish *alpl* loss-of-function line, resembling HPP disease condition.

### Defects in bone mineralization in *alpl* knockout larvae

**Fig. 3:**
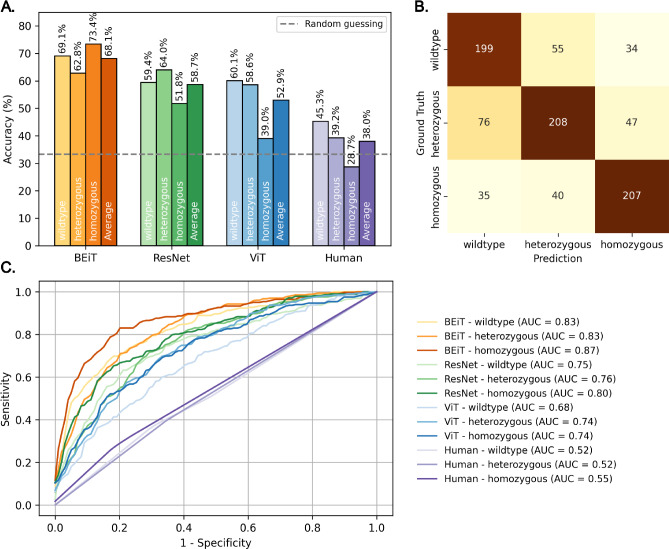
Model performance of different tested AI methods. (**A**) Overall classification accuracy per genotype class for the four methods (BEiT, ResNet, ViT, Human), with the dashed line indicating random-guessing. (**B**) Confusion matrix illustrating the distribution of predictions of the BEiT Model for the three genotype classes. (**C**) Receiver operating characteristic (ROC) curves for each class and method, with corresponding area-under-the-curve (AUC) values.

In HPP patients, loss of TNAP function predominantly results in skeletal malformations and reduced calcification. Therefore, the impact on bone mineralization during craniofacial development of the transgenic *alpl*^wue7^ line was investigated. Bone and cartilage of zebrafish early larvae were stained at 120 hpf with alizarin red and alcian blue (Fig. 2B). This developmental stage was chosen since we wanted to detect changes in bone mineralization as early as possible and bone mineralization starts at 4–5 dpf in zebrafish larvae^50^. In addition, earlier data from TNAP inhibitor experiments on zebrafish larvae at 120 hpf showed strong effects on bone calcification. After staining, individual larvae were dissected into head and tail regions. Heads were further manually dissected into neurocranium and viscerocranium and subsequently imaged. Tails were used for gDNA extraction and subsequent genotyping. To check that the workflow works robustly even with the minor variations seen between independent biological replicates we ensured that this double-staining method was independently performed over seven times from different persons, was imaged by two different microscope systems at different microscope settings (including magnification and intensity levels) and resulted in a data set of 97 investigated individuals and 901 images. Investigation for possible changes in calcification patterns was performed blinded without information about the genotype. Manual image evaluation did not reveal reliable association of distinct mineralization patterns to a certain genotype (Fig. 3A).

For conventional classification, individuals were grouped in “no loss”, “partial loss” and “heavy loss” in bone mineralization. Larvae with “no loss” in mineralization had a fully stained notochord, calcified ceratobranchial 5, and at least two tooth precursor structures, as well as additional visible bone structures, like the parasphenoid or the cleithrum. Larvae with “partial loss” showed a semi-intensely stained notochord with missing staining in the tip of the notochord as well as reduced tooth mineralization with only one visible tooth at each fifth ceratobranchial bow. “Heavy loss” indicated a completely missing red notochord staining (see Table 2). Transgenic *alpl*^wue7^ larvae indicated slight changes in the mineralization of notochord and teeth, but these differences were not consistently distinguishable across all samples and genotypes by simple microscopic observation. 70.8% of the homozygous larvae and 51.3% of the heterozygous larvae showed changed bone mineralization (“partial loss” or “heavy loss”), though similar variations were also observed in 27.7% of the wildtype larvae, making phenotype classification unreliable in a blinded setting (see Table 2). Further image analysis confirmed that also cartilage staining intensity varied greatly between experiments. The high variability in staining patterns across different imaging sessions in addition to HPP phenotype variability and individual experiments suggests that manual phenotype assessment is highly subjective and lacks reproducibility. This motivates the need for automated image-based classification approaches to objectively quantify phenotypic differences in zebrafish skeletal structures.

**Table 2:** Human based classification of bone mineralization loss in 97 stained *alpl*^wue7^ larvae. Larvae with “no loss” in mineralization had a fully stained notochord, at least two stained teeth and additional visible bone structures (like parasphenoid, cleithrum). Larvae with “partial loss” showed a semi-intensely stained notochord. Larvae with “heavy loss” showed no red stained notochord and/or complete loss in tooth staining.

Genotype	No loss	Partial loss	Heavy loss
Wildtype	72.2% (26/36)	19.4% (7/36)	8.3% (3/36)
Heterozygous	51.3% (19/38)	32.4% (12/38)	18.9% (7/38)
Homozygous	29.2% (7/24)	50% (12/24)	20.8% (5/24)

### AI-based classification shows significant genotype–phenotype correlations

To overcome the limitations of manual assessment, we evaluated three deep learning models (BEiT, ResNet, and ViT) for classifying zebrafish phenotypes into genotype classes (wildtype, heterozygous, homozygous) based on skeletal imaging data in zebrafish. Human performance served as a baseline comparator. Full model configurations are provided in the supplement (see Table S 3 & Table S 4).

The performance of the evaluated models is shown in Table 3. It presents classification results across multiple metrics, including accuracy, AUC, F1-scores, precision, sensitivity (TPR) and specificity (FPR). To assess statistical significance, human performance was compared to that of three deep learning models: BEiT, ResNet, and ViT. Among the models, BEiT exhibited the strongest performance across all metrics. It achieved highest accuracy (68.1%), significantly outperforming both humans and other models—ResNet (58.7%) and ViT (52.9%)—with $p<0.0001$ in all comparisons. In addition, BEiT also obtained the highest AUC (84.3%), reflecting its strong discriminative capability of different classes, followed by ResNet (77.0%) and ViT (71.8%). BEiT further led in F1 score (67.9%), precision (69.6%), sensitivity (68.4%), and specificity (84.0%), which indicated balanced and reliable classification performance, with both low false positive and false negative rates. Notably, human performance was relatively low across all metrics, with accuracy (38.0%) and AUC (54.6%), which was close to random guessing. This highlights the value of AI-based support in addressing the inherent difficulty of the task.

Next, we examine class-specific performance across the three genotype categories, namely wildtype, heterozygous, and homozygous to understand model behavior beyond aggregated metrics Fig. 3

Panel A presents the classification accuracy for each genotype class, alongside the average across classes per model. BEiT consistently outperformed all other models across all genotypes. Even though homozygous samples were the most challenging for all other models, BEiT maintained a clear margin on this class, which showed the model successfully captured distinct visual patterns associated with homozygosity.

Panel B displays the confusion matrix for BEiT. Here, the diagonal cells indicate correct classifications. Misclassifications primarily involved heterozygotes that were often confused with both wildtype (76 cases) and homozygous (47 cases). The more frequent confusion suggests that the feature space between wildtype and heterozygous genotypes is less distinct than for the homozygous category. This may reflect underlying phenotypic similarities between wildtype and heterozygous zebrafish, particularly with regard to partially overlapping mineralization patterns.

Receiver operating characteristic (ROC) curves for each model and class, as well as for human performance, are provided in panel C. The ROC curve illustrates how well a model distinguishes between classes across different classification thresholds in a one-vs-all setting, with the area under the curve (AUC) serving as a summary measure of this ability. As in previous evaluations, BEiT achieved the highest AUC scores across all genotype classes, indicating that it not only predicted accurately, but did so with consistent confidence across a range of decision thresholds. Furthermore, the consistently high AUC scores suggest that BEiT effectively separates wildtype, heterozygous, and homozygous larvae without introducing systematic biases toward any genotype.

### Attention-based visualization reveals biologically relevant decision-making in BEiT model

**Fig. 4:**
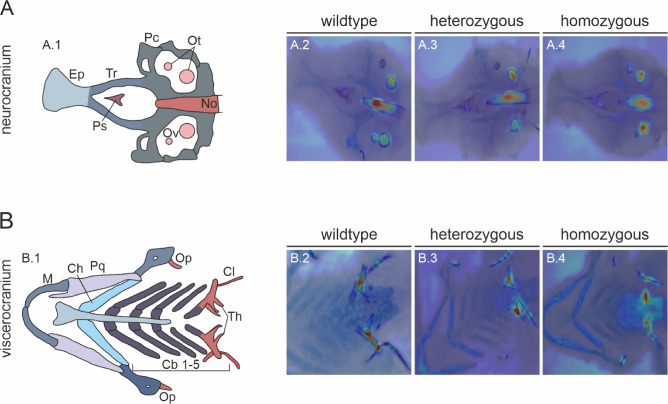
Correlation of AI analyzed structures by attention rollout visualizations overlaid on the original microscopic images. (A.1 & B.1) Annotated illustrations on the left side indicated key structures of the (**A**) neurocranium and (**B**) viscerocranium. (A.2-4 & B.2-4) Right side pictures highlight the spatial distribution of attention across skeletal and cartilaginous structures in different genotype groups. Cb, ceratobranchial cartilage; Ch, ceratohylal cartilage; Cl, cleithrum; Ep, ethmoid plate; M, Meckel’s cartilage; No, notochord; Op, operculum; Ot, otoliths; Ov, otic vesicle; Pc, parachordal cartilage; Pq, palatoquadrate cartilage; Th, teeth; Tr, trabecular cartilage.

To evaluate the interpretability of the BEiT model’s predictions, we conducted attention rollout analysis to identify image regions contributing most to classification decisions. These heatmaps were overlaid on original bone and cartilage staining images for each genotype class, indicating the biological relevance of the investigated areas (Fig. 4). In the neurocranium, the model consistently focused on the notochord and otoliths, which are early-forming mineralized structures. Unlike the notochord, otoliths are not bone tissue, but mineralized parts of the otic vesicle, the early hearing and balancing organ of the zebrafish. The parasphenoid, which is also an early developing skeletal structure, additionally emerged as an important contributor in some cases (Fig. 4A). Within the viscerocranium, the dental papillae of the fifth ceratobranchial arch were the primary focus across all genotypes. The cleithrum was also highlighted in certain cases (Fig. 4B). These earliest bone tissues of the zebrafish align with known sites of TNAP-dependent mineralization, validating the biological relevance of the model’s focus areas. Notably, cartilage elements did not prominently influence classification, which suggested that the AI model primarily relies on bone-specific features. Across all genotypes, similar regions were emphasized. However, in homozygous mutants, a lack of mineralization led to a relative absence of attention signal in the affected areas.

## Discussion

In the following, we first discuss the contributions of this research. Thereafter, we elaborate on the implications resulting from the contributions.

First, our study provides novel insights into the phenotype-genotype correlation in the newly established HPP model. Establishing clear correlations between phenotype and genotype presents challenges not only in our transgenic *alpl*^wue7^ line but also represents a broader issue in patients diagnosed with HPP^12,17^. HPP is a highly heterogeneous, multisystemic disorder characterized by significant phenotypic variability, even among family members sharing the same genetic background^18^. Although numerous studies have addressed this challenge, definitive genotype-phenotype correlations in HPP remain elusive^12,17^.

Using our newly developed AI model, we successfully correlated the phenotype of our *alpl*^wue7^ line with its corresponding genotype, achieving high statistical significance. The accuracy of our AI model strongly indicates that substantial genotype-phenotype correlations exist within HPP. Establishing such correlations was previously unattainable with manual classification carried out by humans. Our findings therefore suggest that HPP might not as heterogeneous as currently assumed; rather, variations in clinical phenotype might be too subtle for humans to identify.

In addition, the visualization of the decision-making process of the AI model using attention rollout provided further insights into the phenotype-genotype correlation. As expected, the model focused on regions associated with early bone development. Interestingly, it also concentrated on the otoliths, calcium carbonate structures within the otic vesicle that develop into the zebrafish’s inner ear^44^. Although the mineralization process of otoliths differs from that of vertebral bone, in which Tnap plays a central role^45^, these structures appear to be altered in our *alpl*^wue7^ line. This may represent a secondary effect of generally disregulated mineralization. Different genetic factors have been identified in zebrafish mutant lines which display prominent otolith malformations , e.g. *stm*: starmaker (stm)^46^ or *pks1*: no content (nco)/corkscrew (csr)/vanished (vns)^47^, but have not been linked to Alpl function yet.

Second, we developed the first image classification AI model capable of correlating the HPP bone phenotype with genotype in zebrafish. Prior research has applied AI in diagnosing HPP within a metabolic context, but did not use image-based methods^48^. By applying deep learning, our study enabled an automated and unbiased analysis of skeletal structures, providing novel insights into the complex phenotypic composition of HPP.

Third, with the generation of our knockout *alpl*^wue7^ zebrafish line, we introduce a new animal model for HPP. By deleting both the *alpl* promoter and start codon, we aimed to eliminate potential off-target effects or genetic compensation mechanisms, which can arise from the expression of defective mRNA, such as that resulting from frameshift mutations^23,24^. As we demonstrated a highly statistically significant downregulation of *alpl* expression in homozygous *alpl*^wue7/wue7^ larvae at 120 hpf, the resulting bone phenotype is expected to accurately reflect the pathological features of severe HPP. Although, this newly established *alpl*^wue7^ line is different to observations made either in HPP patients or classical *Alpl* knockout mouse models, as the heterozygous state in zebrafish is variable. Our observations indicate a rather normal level of *alpl* expression in heterozygous *alpl*^wue7/+^ embryos (see supplement Figure S 3) and inhomogeneous histological observations in this group (see Table 2). Reasons for this discrepancy might be due to technical (e.g. pooled embryos for qPCR analyses) or biological reasons (e.g. compensatory mechanisms) and are currently under investigation. Our newly established AI tool helps us to correlate variable histological changes and genotype also for this group with high reliability.

**Table 3:** Performance metrics comparison for different models, including human performance. Statistical significance was assessed using a Student’s t-test comparing the AI models against human performance. Significance levels: *$p<0.05$, **$p<0.01$, ***$p<0.001$. All values are given in percentage and represent the mean ± standard deviation. The highest value for each metric is bold and underlined, and the second-highest is bold only.

Metric	BEiT	ResNet	ViT	Human
AUC	$\underline{{\textbf {84.3 }}\pm {\textbf {2.0}}}$***	77.0 ± 3.4***	71.8 ± 3.1***	54.6 ± 3.6
Accuracy	$\underline{{\textbf {68.1}} \pm {\textbf {2.3}}}$***	58.7 ± 1.9*	52.9 ± 3.1	38.0 ± 6.4
F1 Score	$\underline{{\textbf {67.9}} \pm {\textbf {2.0}}}$***	58.4 ± 1.9**	51.6 ± 3.9**	36.8 ± 5.3
Precision	$\underline{{\textbf {69.6}} \pm {\textbf {1.8}}}$***	60.5 ± 2.8***	55.4 ± 4.4***	38.2 ± 4.2
TPR	$\underline{{\textbf {68.4}} \pm {\textbf {2.5}}}$***	58.4 ± 1.8**	52.6 ± 3.4**	37.6 ± 5.1
FPR	$\underline{{\textbf {84.0}} \pm {\textbf {1.2}}}$***	79.2 ± 0.9**	76.4 ± 1.6*	68.9 ± 2.7

Fourth, through the generation of our dataset comprising nearly 1000 microscopic images of bone and cartilage double staining in zebrafish, we provide a valuable new resource for the field. This dataset can be used to train AI models, particularly for handling complex and challenging image data. Since zebrafish image classification remains a relatively underexplored area in AI research, the availability of such datasets supports foundational model training and facilitates progress in similar classification tasks.

The aforementioned contributions also have important implications for future research and therapeutic development. First, the implemented AI model offers a robust and unbiased assay for drug screening in the context of HPP. Treated *alpl*^wue7^ larvae can be stained using the same skeletal imaging protocol, and the AI model can then evaluate treatment efficacy by determining whether treated homozygous individuals are phenotypically classified as resembling heterozygous or wildtype fish—indicating a potential rescue effect. This strategy provides a rapid, scalable, and reproducible approach to high-throughput screening, with the potential to significantly accelerate early-phase compound evaluation for HPP. Furthermore, the methodology is broadly applicable and could be adapted for drug discovery in other skeletal disorders exhibiting subtle phenotypic variation.

Second, by applying attention rollout to visualize the AI’s decision-making process, our study provides an example of how explainable AI can advance the field of biomedical imaging. Explainability techniques contribute not only to greater transparency and trust in the AI but also to deeper biological insight—especially in the context of heterogeneous diseases such as HPP. Notably, the model’s unexpected focus on otoliths highlights its ability to detect subtle, clinically relevant features that might otherwise be overlooked. Our results underscore the value of explainable AI for dissecting phenotypic variability, where human interpretation is often limited by cognitive bias based on prior expectations or a narrow focus. In the long term, such interpretable image classification systems hold promise for supporting automated diagnosis, phenotype stratification, and treatment monitoring in clinical practice.

Third, our model offers valuable insights into training AI systems under data-limited conditions. Despite operating in a niche domain with a relatively small dataset, the model achieved high classification performance, demonstrating the effectiveness of data-efficient strategies such as transfer learning and careful dataset curation. These findings highlight best practices for developing robust AI models in biomedical contexts where large, annotated datasets are often unavailable. As such, our approach may serve as a template for other applications in biology and medicine that face similar data constraints.

Finally, given the successful application of AI in our zebrafish HPP model, our approach also holds strong potential for adaptation to other experimental systems, including additional HPP models such as mouse or cell culture-based platforms. Moreover, it could be extended to address phenotypic heterogeneity in other skeletal disorders, such as in osteoporosis zebrafish models^49^, and potentially in broader contexts where subtle morphological variation complicates diagnosis, monitoring, or therapeutic evaluation.

## Supplementary Information

Supplementary Information.

## Data Availability

The dataset supporting the conclusions of this article is included within the article and its additional files. Newly produced materials are available upon request. https://zenodo.org/records/15269595. The code used in the creation of this paper is available on GitHub (https://github.com/simonzrln/zebrafish_paper).
