# Supplementary material for: AI-assisted phenotyping in a zebrafish hypophosphatasia model enables early and precise detection of skeletal alterations
Source: Sci Rep. 2025 Sep 17;15:32578. doi: 10.1038/s41598-025-19199-w (PMC12443964; doi:10.1038/s41598-025-19199-w)
Supplement: Supplementary file 1 — Supplementary Information. [file 41598_2025_19199_MOESM1_ESM.pdf]

Supplementary Material

Supplementary Tables

Primers, that were used for hybridization to generate inserts for the CRISPR target sides are shown in Table S 1.

Table S 1: Primers used for cloning of *alpl* sgRNA target sequences into the pDR274 vector, all sequences are depicted 5´ to 3´

**Target**	**Locus**	**Target sequence**	**Primer forward**	**Primer reverse**
Target 1	*alpl* 5´ UTR	AAACGG<br>CTCCTATC<br>AACAGG	TAGGAAAC<br>GGCTCCTAT<br>CAACAGG	AAACCCTG<br>TTGATAGGA<br>GCCGTTT
Target 2	*alpl* intron 1	ACATGG<br>CTTCCTTG<br>GCATGT	TAGGACAT<br>GGCTTCCTT<br>GGCATGT	AAACACAT<br>GCCAAGGAA<br>GCCATGT

Primers, which were used for PCR amplification of the *alpl* promoter region are shown in Table S 2 together with their sequence, their melting temperature (T_a) and their experimental application.

Table S 2: Primers used for PCR and Sanger Sequencing, sequences are depicted 5´ to 3´

**Primer**	**Sequence**	**T_a(°C)**	**Application**
Zf_alpl_gDNA_5UTR_fwd	GCTACATCCAA GCAACATACCA	57	PCR amplification *alpl* promoter
Zf_alpl_gDNA_In1_rev	AGTGAAGATGAG GCATGCTTTT	57	PCR amplification *alpl* promoter
Zf_alpl_ex2_qPCR_fwd	TCAGTTGTCT GGCACCCTTG	58	qPCR, intron-spanning, located in exon 2
Zf_alpl_ex3_qPCR_rev	CCGCTTCTCT TGCTCTGGAA	58	qPCR, intron-spanning, located in exon 3
Zf_alpl_ex6_qPCR_fwd	CACAACAACG CGAGTGAACC	58	qPCR, intron-spanning, located in exon 6
Zf_alpl_ex7_qPCR_rev	GGGGTACATG CTTCTCCGTC	58	qPCR, intron-spanning, located in exon 7
Zf_eef1a1l1_qPCR_fwd	GCACGGTGAC AACATGCTG	58	qPCR, intron-spanning, endogeneous cDNA control
Zf_eef1a1l1_qPCR_rev	ACCGCTAGCA TTACCCTCCT	58	qPCR, intron-spanning, endogeneous cDNA control

Full model configurations are provided in Table S 3 and Table S 4.

Table S 3: Validation accuracy (%) for different numbers of unfrozen transformer encoder layers (BEiT and ViT) or convolutional blocks (ResNet). Experiments were conducted on a smaller subset of the dataset. BEiT achieved the highest performance with three unfrozen layers, while ResNet and ViT performed best with only the final block/layer unfrozen. Due to early overfitting or resource limitations, unfreezing of further layers was not tested.

**Unfrozen Layers**	**BEiT**	**ResNet**	**ViT**
1	64.85	**61.17**	**55.97**
2	68.91	58.34	53.42
3	**71.64**	–	–
4	65.33	–	–
5	61.42	–	–

Table S 4: Hyperparameter settings for BEiT, ViT, and ResNet101 training

**Hyperparameter**	**BEiT**	**ViT**	**ResNet101**
Model Architecture	BEiT-Large (patch16-512)	ViT-Base (patch16-224)	ResNet101
Input Image Size	512 × 512	512 × 512	512 × 512
Batch Size	32	32	32
Optimizer	AdamW	AdamW	AdamW
Learning Rate	0.001	0.001	0.001
Weight Decay	1×10^{-4}	1×10^{-4}	1×10^{-4}
Maximum Epochs	50	50	30
Early Stopping Patience	5	5	5
Loss Function	Cross-Entropy Loss	Cross-Entropy Loss	Cross-Entropy Loss
Train-Validation Splits	5-Fold Stratified K-Fold	5-Fold Stratified K-Fold	5-Fold Stratified K-Fold
Data Augmentations	Horizontal Flip, Rotation (±30°), Color Jitter (Brightness 0.2, Contrast 0.2, Saturation 0.2, Hue 0.1)	Horizontal Flip, Rotation (±30°), Color Jitter (Brightness 0.2, Contrast 0.2, Saturation 0.2, Hue 0.1)	Horizontal Flip, Rotation (±30°), Color Jitter (Brightness 0.2, Contrast 0.2, Saturation 0.2, Hue 0.1)
Normalization Mean	(0.5, 0.5, 0.5)	(0.5, 0.5, 0.5)	(0.485, 0.456, 0.406)
Normalization Std	(0.5, 0.5, 0.5)	(0.5, 0.5, 0.5)	(0.229, 0.224, 0.225)
Transformer Layers Finetuned	Last three encoder layers (21-23), Pooler, Classifier	Last encoder layer (11), LayerNorm, Classifier	Last ResNet block (layer4), Fully Connected Layer (fc)
Gradient Clipping	Not used	0.5	Not used
Number of Workers (DataLoader)	4	4	4

Supplementary Methods

Zebrafish Husbandry

Zebrafish (*Danio rerio*) were kept under standard laboratory conditions in the aquatic facility of the Biocenter of the Julius-Maximilian-University of Würzburg, Germany according to FELASA guidelines [1, 2]. Zebrafish were fed with a mixture of live food (*Artemia nauplii* for adult fish, *Paramecium caudatum* for larvae) and GEMMA Micro Food (size according to their age; Skretting, USA). All animal experimentation has been performed according to the guidelines of the German animal welfare law and approved by the local government (Government of Lower Franconia; Tierschutzgesetz §11, Abs. 1, Nr. 1; husbandry permit number 55.2.2-2532-2-1682; fin clipping permit number 55.2.2-2532-2-1637, HPP generation and husbandry permit number 55.2.2-2532-2-1472). For line generation, the wildtype strain AB/AB (ZFIN ID: ZDB-GENO-960809-7) was used. Zebrafish embryos were kept in 0.30 Daneau´s medium (17.4 mM NaCl, 0.21 mM KCl, 0.12 mM $MgSO_4$, 0.18 mM $Ca(NO_3)_2$, 1.5 mM HEPES, 0.0001 % Methylene blue, pH 7.2) at 28.5 °C and divided into developmental stages based on their morphological characteristics according to Kimmel et al. [3]. "hpf" and "dpf" indicate embryonic development in hours/days post fertilization. If embryos lacking pigment cells were required, they were incubated in Daneau´s medium + 0.003 % 1-phenyl 2-thiourea (PTU; Sigma-Aldrich, USA) for the inhibition of melanogenesis [5].

gRNA Design and Isolation

To generate the *alpl* transgenic zebrafish line, the corresponding promoter targeting sgRNAs were designed as previously described using the CHOPCHOP CRISPR design tool (https://chopchop.cbu.uib.no/) [4]. For the generation of a transgenic *alpl* promoter deletion zebrafish line, two targets in the *alpl* locus were chosen 420 bp upstream and 550 bp downstream of *alpl* transcription start site. Target site 1 (AAACGGCTCCTATCAACAGG) is located to the 5´UTR and target site 2 (ACATGGCTTCCTTGGCATGT) is located in intron 1. Forward and reverse primers were annealed according to their target sequence (see Table S 1) and then cloned into the linearized pDR274 (Addgene #42250) plasmid [6]. After confirmation by Sanger Sequencing, the cloned pDR274 *alpl* sgRNA vectors were linearized by DraI enzyme (#R0129S, New England Biolabs, USA) and the templates were used for *in vitro* RNA transcription via the MAXIscript T7 kit (Ambion/ life technologies, Germany). The gRNAs were purified by phenol-chloroform extraction.

Phenol Chloroform Extraction

gRNAs were purified by phenol-chloroform extraction to remove enzymes, lipids and other contaminants. The RNA samples were adjusted to a total volume of 200 μl with H_2O after *in vitro* transcription. Then, 20 μl of 3 M sodium acetate pH 5.2 was added. 200 μl of a 1:1 mixture of ROTI Aqua-Phenol

and chloroform was mixed to the reaction tube and was centrifugated (12,000 xg, 5 min, 4°C). The aqueous phase was transferred into a new reaction tube, and 200 μl chloroform was added again. Following another centrifugation step (12,000 xg, 5 min, 4 °C), the aqueous phase was transferred again. The gRNA was precipitated by adding 2 volumes of ethanol (absolute, -20 °C). After the incubation (overnight, -20 °C), the RNA was pelleted by centrifugation (21,000 xg, 20 min, 4 °C). The supernatant was removed, and the RNA pellet was rinsed with 500 μl of ethanol (70 %, 4 °C) and again pelleted (21,000 xg, 10 min, 4 °C). The liquid was completely removed, then the pellet was air dried. The gRNA was resuspended in 25 μl H_2O.

Microinjection

One-cell stage zebrafish embryos were microinjected using the FemtoJet 4i (Eppendorf, Germany). For the generation of *alpl* CRISPR lines, a solution comprising of gRNA (200 ng/μl each), EnGen Spy Cas9 NLS protein (250 ng/μl; #M0646T, New England Biolabs, USA), Phenol red (0.05 % [v/v], pH7.0; for visualization of the injection solution) and FITC-dextran (1 mg/μl, Sigma-Aldrich, USA). The solution was incubated (15 min, 37 °C) prior to injection to enable the formation of a gRNA-Cas9 complex. The injection volume was adjusted to approximately 1/10 of cell volume (corresponding to 1.7 nl/embryo). Positively injected embryos were identified by transient green fluorescence of FITC-dextran at 24 hpf and were selected and raised for transgenic line generation.

Establishment of Transgenic Zebrafish Line

In order to generate the $alpl_{\text{wue7}}$ transgenic zebrafish line, sgRNA/Cas9 protein injected larvae were raised as potential founder animals to adulthood (F0 generation). Single F0 animals were subsequently used in crossings to *AB* wildtype zebrafish to generate potential heterozygous transgenic F1 siblings. Positive founder crossings were identified by whole embryo genotyping (8 larvae per batch), while fin-clip genotyping was used to identify adult transgenic F1 individuals. Homozygous larvae were created by incrossing of transgenic zebrafish and were genotyped at 120 hpf by gDNA isolation using protein kinase K digestion and ethanol precipitation.

Genotyping was routinely done via PCR and gel electrophoresis for individuals showing gDNA deletions. Optionally, Sanger Sequencing was conducted for validation of genetic changes (see Table S 2 for used primers). For sequencing, PCR products were amplified spanning the targeted genomic region and background signals were removed using an ExoSAP kit (biotechrabbit GmbH, Germany). Then, the DNA fragments were prepared for sequencing using the BigDye Terminator v3.1 Cycle Sequencing Kit (Thermo Fisher Scientific, UK). The reactions were purified prior to sequencing by sodium acetate precipitation. Sanger sequencing was performed using the 3130xl Genetic Analyzer or the 3730

Genetic Analyzer (Applied Biosystems, USA). The obtained sequences were analyzed by the software ApE (https://jorgensen.biology.utah.edu/wayned/ape/) and CodonCode Aligner.

RNA Isolation of Genotyped Larvae, cDNA Synthesis and Quantitative Real-Time PCR (qPCR)

For investigation of *alpl* mRNA expression in the *alpl*$^{\mathrm{wue7}}$ zebrafish line, we performed qPCR experiments on cDNA of pooled larvae according to their genotype. At first, living zebrafish larvae were genotyped at 4 dpf according to an early fin clipping protocol [7]. Following a microscopic fin biopsy , DNA was extracted with a NaOH based protocol and larvae could be genotyped by PCR (primer see Table S 2). At 120 hpf, larvae were collected, pooled according to their genotype (wildtype, heterozygous and homozygous) and frozen at -80°C. Each sample contained 9-15 whole zebrafish larvae, for each genotype, three pools were collected. RNA was isolated using the Monarch total RNA Miniprep Kit (#T2010S; New England Biolabs, USA) according to manufacturers protocol including a DNase I treatment. 1 μg total RNA was used for reverse transcription, which was performed using the FIREScript RT cDNA synthesis KIT (Solis BioDyne, Estonia) according to manufacturer´s instructions. Transcript level of *alpl* was analyzed by qPCR according to standard methods using the HOT Fire Pol Eva Green qPCR Mix Plus (Solis BioDyne, Estonia). For analysis of *alpl*, primer pairs targeting different exon regions of the *alpl* mRNA were used, and amplification of *eef1a1l1* mRNA was used as a housekeeping gene for endogenous cDNA control (specific primer sequences see Table S 2). The qPCR reactions were performed in technical and biological triplicates on a single plate and further analyzed in a ViiA7 Real-Time PCR System (Thermo Fisher, Scientific, USA). After synthesis, the cDNA was diluted 1:4 in H_2O and 0.25 μl were used per sample. 6.25 μl H_2O, 2 μl 5x HOT FirePol Eva Green Supermix (Solis BioDyne, Estonia), and 1 μl forward and reverse primer mix (2.5 pmol/μl) were added. The PCR program for amplification was 95 °C for 15 min , followed by 40 cycles at 95 °C for 15 sec, 58 °C for 20 sec, and 72 °C for 20 sec. The melting curve was generated using temperatures from 58 °C until 95 °C. Data analysis was performed using the QuantStudio Real-Time PCS Software v1.1 (Thermo Fisher Scientific, USA) by $\Delta\Delta$Ct method and Excel (Microsoft, USA). For each experiment, the according wildtype sample served as reference for the comparison of relative *alpl* gene expression.

Transfer Learning

Transfer learning is a machine learning approach in which a model trained on one task is adapted, either by using its learned representations as fixed features or by finetuning parts of the network for a related target task. In medical imaging, this technique has become one of the most practical paradigms in deep learning, particularly when transferring knowledge from natural images to medical images [8, 9]. It addresses the challenge of data scarcity while also reducing

computational demands [10, 11]. In practice, transfer learning is implemented by initializing a model with pre-trained weights and adapting it to the target task. A common strategy involves layer freezing, where the early layers of the network responsible for extracting general features such as edges and textures are kept unchanged, while the later layers are trainable to learn task specific representations. The number of trainable layers depends on the similarity between the source and target domains. Since deeper layers capture increasingly task specific features, progressively more layers must be unfrozen as the difference between the source and target domains grows.[10, 12, 13]

Layer freezing Strategy

To preserve the general pattern recognition capabilities of the pretrained beit-large-patch16-512 model, the majority of network layers were kept fixed during training. Only the final layers, which contribute most substantially to learning task-specific patterns [12, 13], were allowed to adapt to the new data. To determine the optimal number of layers to remain trainable, a systematic search strategy [14] was applied to a smaller subset of the training data.

Supplementary Data and Figures

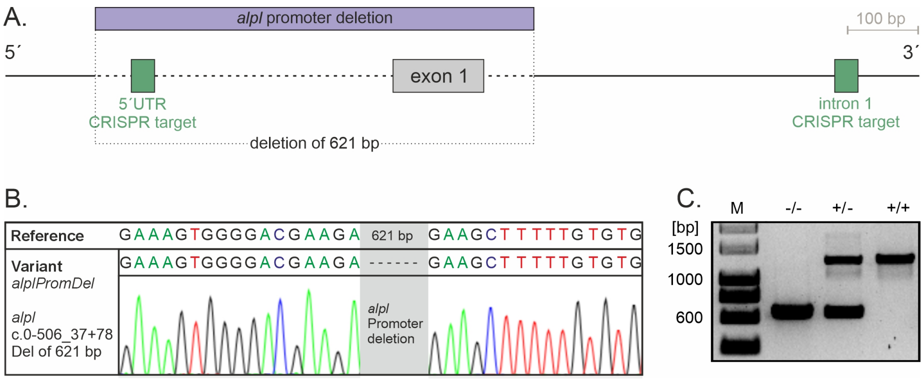

Figure S 1: **Visualization of genetic alterations in the transgenic *alpl*$^{\mathbf{wue7}}$ zebrafish line.** (A) Schematic depiction of the *alpl* promoter region flanking exon 1 (gray box), including 5´UTR and intron 1 CRISPR target sites (green boxes). The deleted region in *alpl*$^{\text{wue7}}$ fish is marked with a dashed line and comprises of 621 bp. Scale bar indicates distance of 100 bp. (B) Validation of *alpl* promoter deletion via Sanger Sequencing on single embryo. Missing reference sequence is grayed out and includes the missing 621 bp. (C) PCR amplification of single embryo *alpl* promoter region showed PCR band size variations. Homozygous and heterozygous samples display loss of the 621 bp in transgenic *alpl* zebrafish embryos. (M = marker; -/- = *alpl*$^{\text{wue7/wue7}}$; -/+ = *alpl*$^{\text{wue7/+}}$; +/+ = *alpl*$^{+/+}$). Full gel image see Figure S 2.

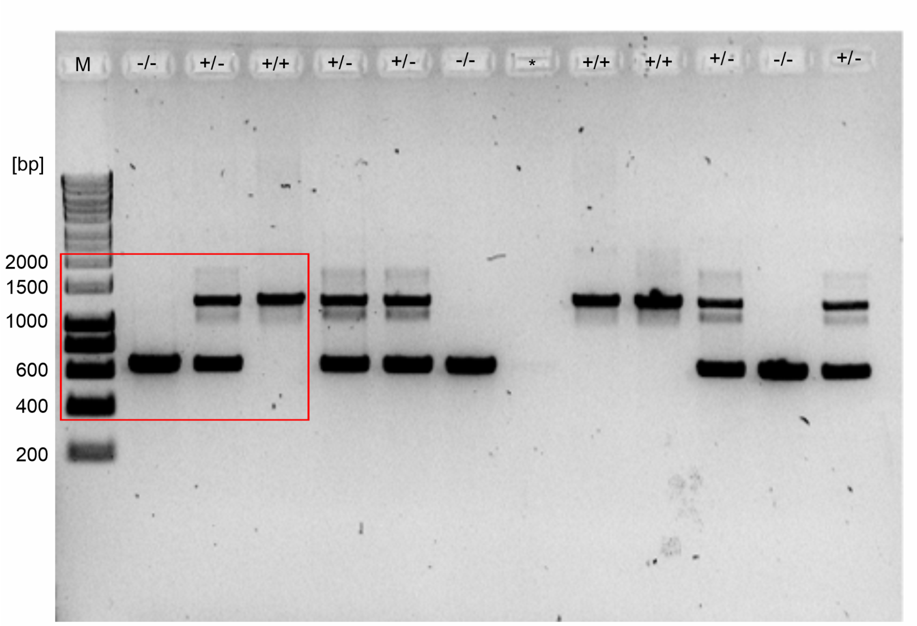

Figure S 2: **Full gel image of *alpl*$^{\mathbf{wue7}}$ zebrafish line.** Genomic DNA PCR amplification of single embryo *alpl* promoter region showed PCR band size variations. Homozygous and heterozygous samples display loss of the 621 bp in transgenic *alpl* zebrafish embryos. The red box marks the cropped area shown in Figure S 1 C. (M = 1 kb DNA HyperLadder (Meridian Bioscience, Inc.; USA); -/- = *alpl*$^{wue7/wue7}$; -/+ = *alpl*$^{wue7/+}$; +/+ = *alpl*$^{+/+}$; * = control PCR without DNA insert)

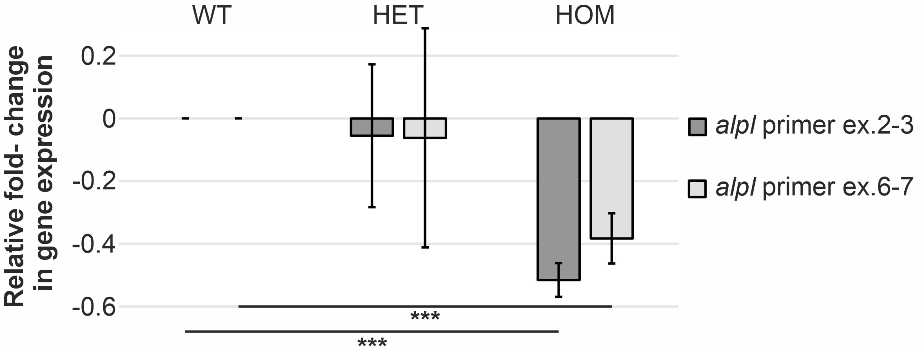

Figure S 3: ***alpl* expression in the transgenic *alpl*$^{\mathbf{wue7}}$ line.** Expression of *alpl* mRNA in the three genotypic classes, wildtype (WT), heterozygous (HET), and homozygous (HOM) was determined at mRNA level with qPCR on cDNA from whole embryos at 120 hpf. Each genotype was tested in a biological triplicate (n=3) and additionally in a technical triplicate (n=3). The relative fold-change in gene expression was determined using the $2^{-\Delta\Delta Ct}$ method. This value is depicted in a logarithmic manner. Two cDNA loci were covered using the two primer pairs ex.2-3 and ex.6-7. All samples were normalized to the corresponding wildtype samples of each experiment. Error bars correspond to the standard deviation. Statistics were calculated using a two-sited Welch´s t-test, *** $p < 0.001$. Heterozygous larvae did not indicate a significant *alpl* transcript reduction. In homozygous larvae, the *alpl* transcript reduction is highly statistically significant for both primer pairs.

References

[1] Westerfield, M. *The Zebrafish Book: A Guide for the Laboratory Use of Zebrafish (Danio Rerio)* (University of Oregon Press, 2000). URL https://books.google.de/books?id=Iy8PngEACAAJ.

[2] Aleström, P. *et al.* Zebrafish: Housing and husbandry recommendations. *Laboratory Animals* **54**, 213–224 (2020). URL https://journals.sagepub.com/doi/abs/10.1177/0023677219869037https://pmc.ncbi.nlm.nih.gov/articles/PMC7301644/pdf/10.1177_0023677219869037.pdf.

[3] Kimmel, C. B., Ballard, W. W., Kimmel, S. R., Ullmann, B. & Schilling, T. F. Stages of embryonic development of the zebrafish. *Dev Dyn* **203**, 253–310 (1995).

[4] El-Brolosy, M. A. *et al.* Genetic compensation triggered by mutant mrna degradation. *Nature* **568**, 193–197 (2019).

[5] Karlsson, J., von Hofsten, J. & Olsson, P. E. Generating transparent zebrafish: a refined method to improve detection of gene expression during embryonic development. *Mar Biotechnol (NY)* **3**, 522–7 (2001).

[6] Hwang, W. Y. *et al.* Efficient genome editing in zebrafish using a crispr-cas system. *Nat Biotechnol* **31**, 227–9 (2013).

[7] Kosuta, C. *et al.* High-throughput dna extraction and genotyping of 3dpf zebrafish larvae by fin clipping. *J Vis Exp* (2018).

[8] Zhou, Z., Sodha, V., Pang, J., Gotway, M. B. & Liang, J. Models genesis. *Medical image analysis* **67**, 101840 (2021).

[9] Zhang, W. *et al.* Deep model based transfer and multi-task learning for biological image analysis. In *Proceedings of the 21th ACM SIGKDD International Conference on Knowledge Discovery and Data Mining*, 1475–1484 (2015).

[10] Zhuang, F. *et al.* A comprehensive survey on transfer learning. *Proceedings of the IEEE* **109**, 43–76 (2020).

[11] Pan, S. J. & Yang, Q. A survey on transfer learning. *IEEE Transactions on knowledge and data engineering* **22**, 1345–1359 (2009).

[12] Yosinski, J., Clune, J., Bengio, Y. & Lipson, H. How transferable are features in deep neural networks? *Advances in neural information processing systems* **27** (2014).

[13] Gerace, F., Doimo, D., Mannelli, S. S., Saglietti, L. & Laio, A. Optimal transfer protocol by incremental layer defrosting. *arXiv preprint arXiv:2303.01429* (2023).

[14] Liashchynskyi, P. & Liashchynskyi, P. Grid search, random search, genetic algorithm: A big comparison for nas (2019). URL https://arxiv.org/abs/1912.06059. 1912.06059.
